# Influences of amyloid-β and tau on white matter neurite alterations in dementia with Lewy bodies

**DOI:** 10.1038/s41531-024-00684-4

**Published:** 2024-04-03

**Authors:** Elijah Mak, Robert I. Reid, Scott A. Przybelski, Timothy G. Lesnick, Christopher G. Schwarz, Matthew L. Senjem, Sheelakumari Raghavan, Prashanthi Vemuri, Clifford R. Jack, Hoon Ki Min, Manoj K. Jain, Toji Miyagawa, Leah K. Forsberg, Julie A. Fields, Rodolfo Savica, Jonathan Graff-Radford, David T. Jones, Hugo Botha, Erik K. St. Louis, David S. Knopman, Vijay K. Ramanan, Dennis W. Dickson, Neill R. Graff-Radford, Tanis J. Ferman, Ronald C. Petersen, Val J. Lowe, Bradley F. Boeve, John T. O’Brien, Kejal Kantarci

**Affiliations:** 1 Department of Radiology, Mayo Clinic, Rochester, MN USA; 2 Department of Psychiatry, University of Cambridge, Cambridge, UK; 3 Department of Information Technology, Mayo Clinic, Rochester, MN USA; 4 Department of Quantitative Health Sciences, Mayo Clinic, Rochester, MN USA; 5 Department of Radiology, Mayo Clinic, Jacksonville, FL USA; 6 Department of Neurology, Mayo Clinic, Rochester, MN USA; 7 Department of Psychiatry and Psychology, Mayo Clinic, Rochester, MN USA; 8 Center for Sleep Medicine, Division of Pulmonary and Critical Care Medicine, Department of Medicine, Mayo Clinic, Rochester, MN USA; 9 Department of Psychiatry and Psychology, Mayo Clinic, Jacksonville, FL USA; 10 Laboratory of Medicine and Pathology, Mayo Clinic, Jacksonville, FL USA; 11 Department of Neurology, Mayo Clinic, Jacksonville, FL USA

**Keywords:** Biophysical models, Dementia

## Abstract

Dementia with Lewy bodies (DLB) is a neurodegenerative condition often co-occurring with Alzheimer’s disease (AD) pathology. Characterizing white matter tissue microstructure using Neurite Orientation Dispersion and Density Imaging (NODDI) may help elucidate the biological underpinnings of white matter injury in individuals with DLB. In this study, diffusion tensor imaging (DTI) and NODDI metrics were compared in 45 patients within the dementia with Lewy bodies spectrum (mild cognitive impairment with Lewy bodies (*n* = 13) and probable dementia with Lewy bodies (*n* = 32)) against 45 matched controls using conditional logistic models. We evaluated the associations of tau and amyloid-β with DTI and NODDI parameters and examined the correlations of AD-related white matter injury with Clinical Dementia Rating (CDR). Structural equation models (SEM) explored relationships among age, APOE ε4, amyloid-β, tau, and white matter injury. The DLB spectrum group exhibited widespread white matter abnormalities, including reduced fractional anisotropy, increased mean diffusivity, and decreased neurite density index. Tau was significantly associated with limbic and temporal white matter injury, which was, in turn, associated with worse CDR. SEM revealed that amyloid-β exerted indirect effects on white matter injury through tau. We observed widespread disruptions in white matter tracts in DLB that were not attributed to AD pathologies, likely due to α-synuclein-related injury. However, a fraction of the white matter injury could be attributed to AD pathology. Our findings underscore the impact of AD pathology on white matter integrity in DLB and highlight the utility of NODDI in elucidating the biological basis of white matter injury in DLB.

## Introduction

Dementia with Lewy bodies (DLB) is the second most prevalent cause of dementia in the elderly after Alzheimer’s disease (AD) and is characterized by core symptoms, including visual hallucinations, parkinsonism, cognitive fluctuations, and rapid eye movement (REM) sleep behavior disorder^1^. Neuropathologically, DLB is defined by the accumulation of α-synuclein and the formation of Lewy bodies and Lewy neurites, although autopsy studies have revealed that up to 70% of individuals with DLB also exhibit co-existing extracellular amyloid-β plaques and intracellular paired helical filaments of tau, both of which negatively impact disease progression, prognosis, and survival^2–4^.

While it is widely accepted that amyloid accumulation and tau pathology play a crucial role in the disease severity and progression of DLB^5^, the precise mechanisms underlying their contributions to the clinical phenotypes and disease progression in DLB remain elusive. Post-mortem studies have demonstrated that DLB patients with high Braak neurofibrillary tangle (NFT) tau stages exhibit more severe atrophy in stereotypical AD regions, such as the temporoparietal cortices, hippocampus, and amygdala^6–8^, and these findings have since been corroborated by in vivo positron emission tomography (PET) imaging studies^9–12^. Although the aforementioned associations between AD pathologies and gray matter atrophy point towards a potential influence on the clinical heterogeneity and disease progression through neurodegeneration, the sensitivity and specificity of gray matter biomarkers to detect incipient AD-related pathological insults may still be limited due to the numerous cellular changes that may precede macroscale neuronal loss, such as microstructural injury within the white matter bundles^13–15^.

Diffusion tensor imaging (DTI) has been extensively utilized in numerous studies to characterize deficits of white matter integrity and other microstructural injury in DLB^16,17^. These studies have consistently revealed reduced fractional anisotropy (FA) and increased mean diffusivity (MD) primarily in the parieto-occipital white matter tracts^17–19^. However, our understanding of the pathological drivers of white matter injury in DLB remains limited due to the lack of data on the contribution of AD pathologies to DTI deficits, despite growing post-mortem evidence^20^ and recent in vivo data implicating tau in spatially dependent white matter deficits^21^. The interpretation of FA and MD is further complicated by their sensitivity to multiple microstructural injury, not least including axonal loss, demyelination, and gliosis^22^. Additionally, partial volume effects and crossing fibers, as well as the presence of free water in the extracellular space, could lead to an overestimation of MD and an underestimation of FA.

Recent biophysical models such as Neurite Orientation Dispersion and Density Imaging (NODDI) can differentiate signals from multiple tissue compartments, including intra- and extracellular spaces, to derive quantitative estimates of neurite density and orientation dispersion (i.e., angular variation in fiber orientations)^23^. As such, the NODDI model can provide biologically specific insights into the cellular changes (e.g., axonal loss in white matter tracts) that may underlie the abnormalities seen in conventional DTI techniques^18^. Indeed, several studies using NODDI have delineated relevant neurobiological changes in normal aging^24–26^, young-onset AD^27^, and primary tauopathies^28^.

**Fig. 1:**
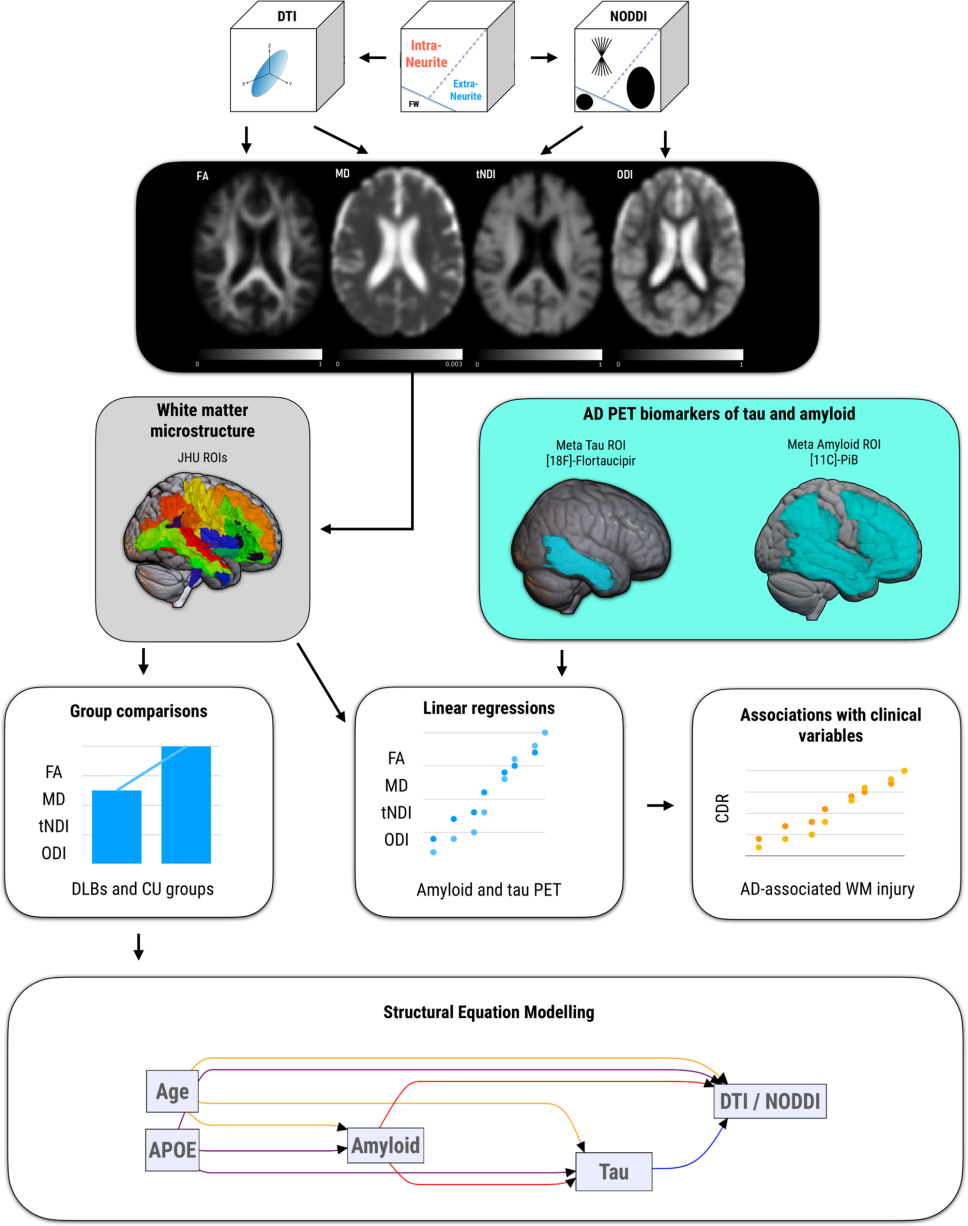
Overview of the study aims and analyses. The study design involved fitting diffusion-weighted imaging datasets with DTI and NODDI models to obtain whole-brain maps of FA, MD, tNDI, and ODI. These maps were then parcellated using the JHU “Eve” atlas to derive bilateral median values across 40 ROIs. We employed conditional logistic models to compare regional DTI and NODDI metrics between the DLBs and CU groups while using regressions to assess the topographical associations of [18 F]-Flortaucipir and [11 C]-PiB uptake with regional DTI and NODDI metrics, adjusting for age and APOE genotype. Statistical results were corrected for multiple comparisons using FDR. Partial Pearson’s correlations were used to determine the degree to which AD-related white matter injury is associated with CDR-SOB, adjusting for age. Statistically significant ROIs from the group-wise comparisons were used as composite outcome measures in SEMs to explore multivariable relationships among age, APOE genotype, amyloid, tau, and white matter injury in the DLBs. Abbreviations: AD Alzheimer’s disease, APOE Apolipoprotein E, CU cognitively unimpaired, DLBs Dementia with Lewy bodies spectrum, CDR-SOB Clinical Dementia Rating - Sum of Boxes, PiB Pittsburgh compound B, DTI Diffusion tensor imaging, NODDI Neurite Orientation Dispersion and Density Imaging, FDR False Discovery Rate, FA Fractional anisotropy, MD Mean diffusivity, tNDI tissue-weighted Neurite Density Index, ODI Orientation Dispersion Index, ROIs Regions of Interest, SEM.

To date, no study has characterized the regional distributions of NODDI parameters in DLB or delineated the in vivo topographical correlations of white matter microstructural injury with PET biomarkers of amyloid-β and tau. We addressed these gaps by (1) Comparing the regional distributions of DTI and NODDI parameters between individuals with DLBs and matched clinically unimpaired (CU) controls; (2) investigating the topographical associations of white matter integrity with cortical [11 C]-PiB and [18 F]-Flortaucipir uptake in DLBs to identify the white matter tracts most closely associated with AD-related injury; (3) assessing the degree to which AD-related white matter injury is related to clinical disease severity; (4) and evaluating the multivariable associations of age, APOE genotype, cortical [11 C]-PiB and [18 F]-Flortaucipir uptake with DTI and NODDI parameters using Structural Equation Models (SEM). An overview of our study design is illustrated in Fig. 1.

## Results

### Sample characteristics

Participant characteristics are summarized in Table 1. By design, the DLBs and CU groups were comparable in terms of age, with an average age of 68.8 ± 9.2 years (*p* = 0.27). There were no statistically significant differences between the groups in terms of sex distribution, education years, or *APOE* status. As expected, the DLBs had statistically significantly lower MMSE scores (23.5 ± 5.3) than the CU group (29.2 ± 0.8; *p* < 0.001; conditional logistic regressions). Cortical [18 F]-Flortaucipir SUVr was statistically significantly higher in DLBs (1.27 ± 0.21) compared to the CU group (1.19 ± 0.09; *p* = 0.016; conditional logistic regressions). Furthermore, abnormal [18 F]-Flortaucipir uptake was observed in 42% of individuals on the DLB spectrum, compared to 22% of CU participants (*p* = 0.027; conditional logistic regressions). Similarly, cortical [11 C]-PiB SUVr was statistically significantly higher in the DLBs group (1.73 ± 0.45) than in the CU group (1.50 ± 0.33; *p* = 0.010; conditional logistic regressions), and abnormal PiB uptake was observed in 56% of individuals with DLBs, compared to 27% of CU individuals (*p* = 0.009; conditional logistic regressions). Regarding clinical DLB features, 42% of patients with DLBs had visual hallucinations, 64% had cognitive fluctuations, 91% had parkinsonism, and 93% had RBD.

**Table 1:** Sample characteristics

Variables	CU	DLBs	*P*-value
Age, years	68.8 (9.2)	68.8 (9.2)	0.27
Males, no. (%)	39 (87%)	39 (87%)	1.00
APOE, no. (%)	11 (27%)	20 (47%)	0.10
Education, years	15.7 (2.3)	15.6 (2.9)	0.87
MMSE	29.2 (0.8)	23.5 (5.3)	<0.001
CDR-SOB	0.0 (0.0)	5.4 (3.8)	<0.001
Cortical [18 F]-Flortaucipir SUVr	1.19 (0.09)	1.27 (0.21)	0.016
Abnormal Tau, no. (%)	10 (22%)	19 (42%)	0.027
Cortical [11 C]-PiB SUVr	1.50 (0.33)	1.73 (0.45)	0.010
Abnormal PiB, no. (%)	12 (27%)	25 (56%)	0.009
Visual Hallucinations, no. (%)		19 (42%)	
Fluctuations, no. (%)		29 (64%)	
Parkinsonism, no. (%)		41 (91%)	
RBD, no. (%)		42 (93%)	

*APOE* Apolipoprotein E, *CU* cognitively unimpaired, *DLBs* dementia with Lewy bodies spectrum, *CDR-SOB* Clinical Dementia Rating – Sum of Boxes, *MMSE* Mini-Mental State Examination, *PiB* Pittsburgh compound B, *RBD* Rapid eye movement sleep behavior disorder, *SUVr* Standardized uptake value ratio.

**Fig. 2:**
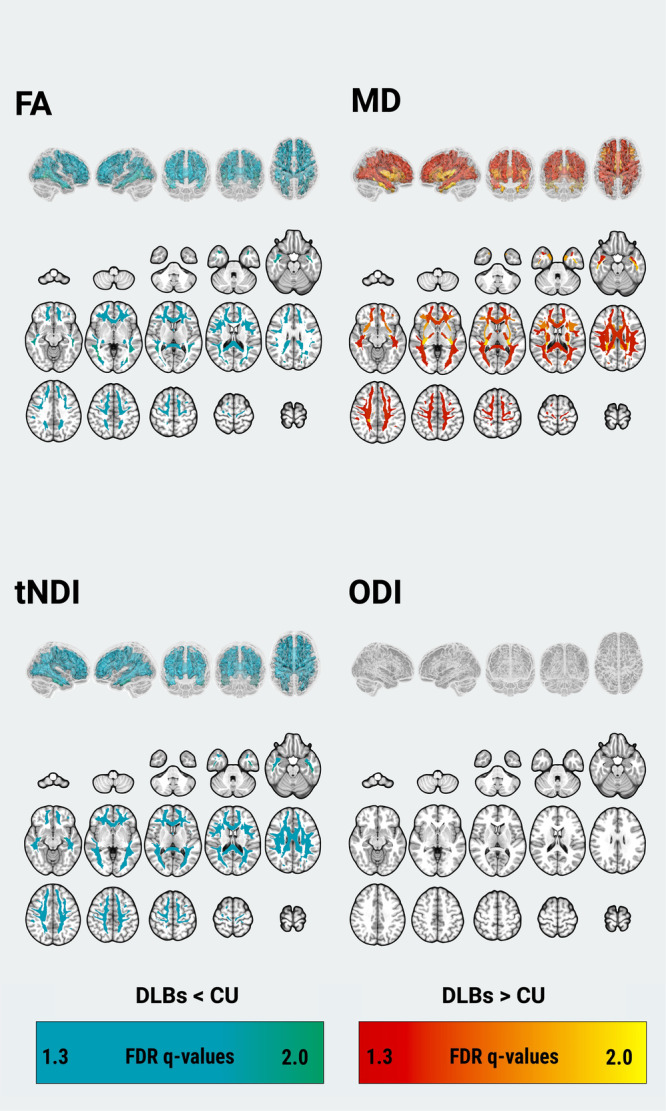
Statistically significant pairwise differences between DLBs and CU in regional FA, MD, tNDI, and ODI are depicted as 3D projections of white matter tracts on glass brain renderings and overlaid on the volumetric MNI152 template. FDR *q* values are expressed as -log10q (i.e., 1.3 = *q* < 0.05). Abbreviations: DLBs Dementia with Lewy bodies spectrum, CU Cognitively unimpaired, FDR False Discovery Rate, FA Fractional anisotropy, MD Mean diffusivity, tND tissue-weighted Neurite Density Index, ODI Orientation Dispersion Index, MNI152Montreal Neurological Institute.

Topographical patterns of between-group differences of DTI and NODDI parameters are illustrated in Fig. 2 and forest plots of group medians (IQRs) and the FDR q values are provided in Supplementary Fig. 1. Compared to the CU group, there were widespread patterns of lower FA (18 ROIs) and higher MD (30 ROIs). In addition, NODDI revealed lower tNDI in DLBs across 24 tracts compared to the CU group. However, there were no statistically significant group differences concerning regional ODI. To evaluate the topological similarity in terms of group differences, we computed Dice coefficient indices between the binarized spatial patterns of statistically significant group differences for FA, MD, and tNDI: (1) FA and MD (Dice = 0.62), (2) FA and tNDI (Dice = 0.71), (3) MD and tNDI (Dice = 0.89). Our findings suggest that the spatial pattern of group differences between FA and MD were moderately similar, while the extent of both FA and MD deficits overlapped strongly with that of the tNDI reductions.

### Associations of AD biomarkers with white matter metrics

**Fig. 3:**
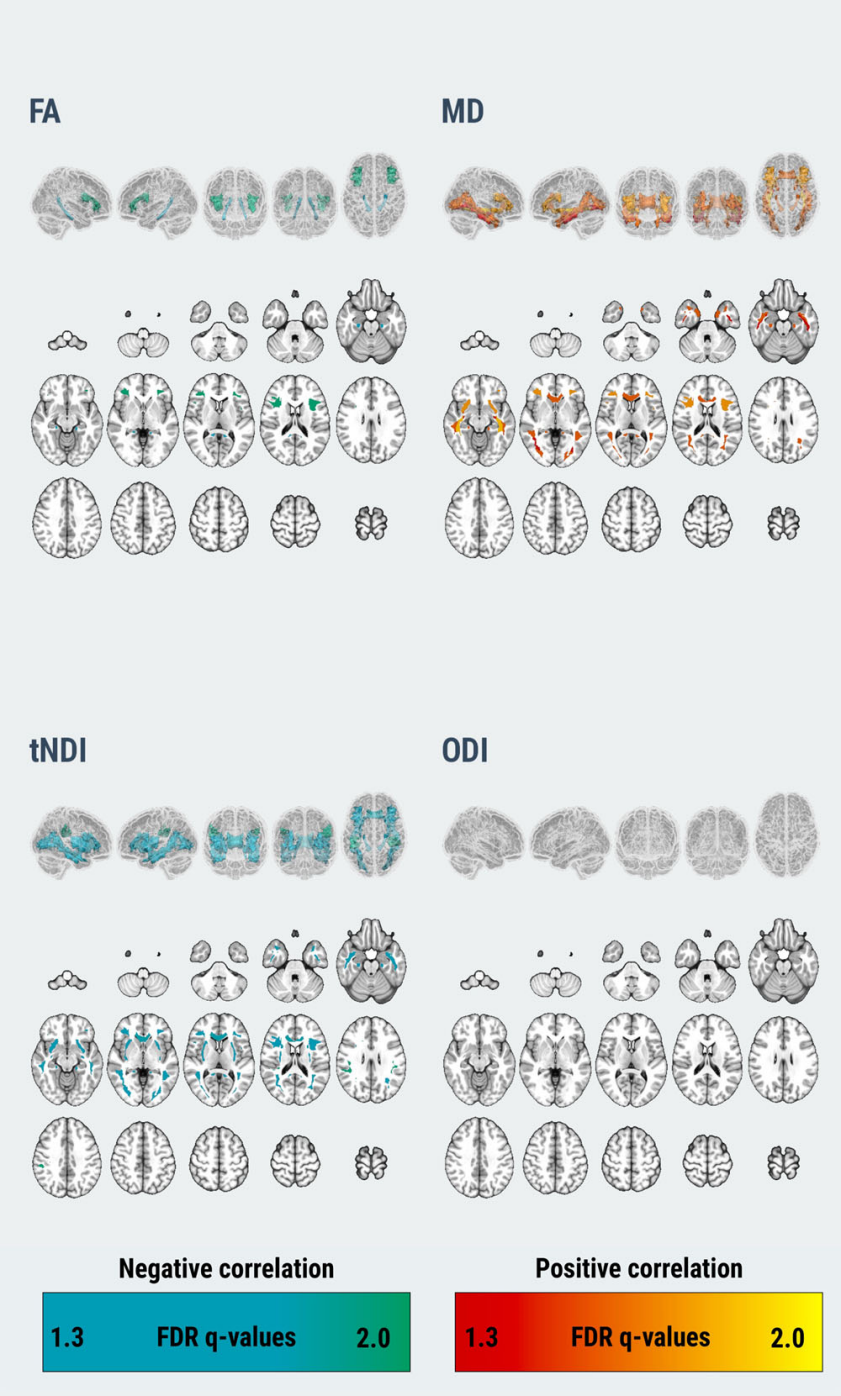
Relationship between tau and white matter microstructure in DLBs. Statistically significant associations of cortical [18 F]-Flortaucipir with regional DTI and NODDI parameters are depicted on 3D glass brain renderings and in volumetric MNI152 space (radiological orientation) after correction for multiple comparisons with FDR. Linear regression models were adjusted for age, APOE genotype, and [11 C]-PiB, and corrected for multiple comparisons with FDR. FDR *q*-values are expressed as -log(q) (i.e., 1.3 -\> *q* = 0.05). Abbreviations: DLBs Dementia with Lewy bodies spectrum; DTI Diffusion tensor imaging, APOE Apolipoprotein E, FDR False Discovery Rate, FA Fractional anisotropy, MD Mean diffusivity, NODDI Neurite Orientation Dispersion and Density Imaging, tNDI tissue-weighted Neurite Density Index, ODI Orientation Dispersion Index; MNI152 Montreal Neurological Institute, SUVr Standardized uptake value ratio, PiB Pittsburgh Compound B.

**Fig. 4:**
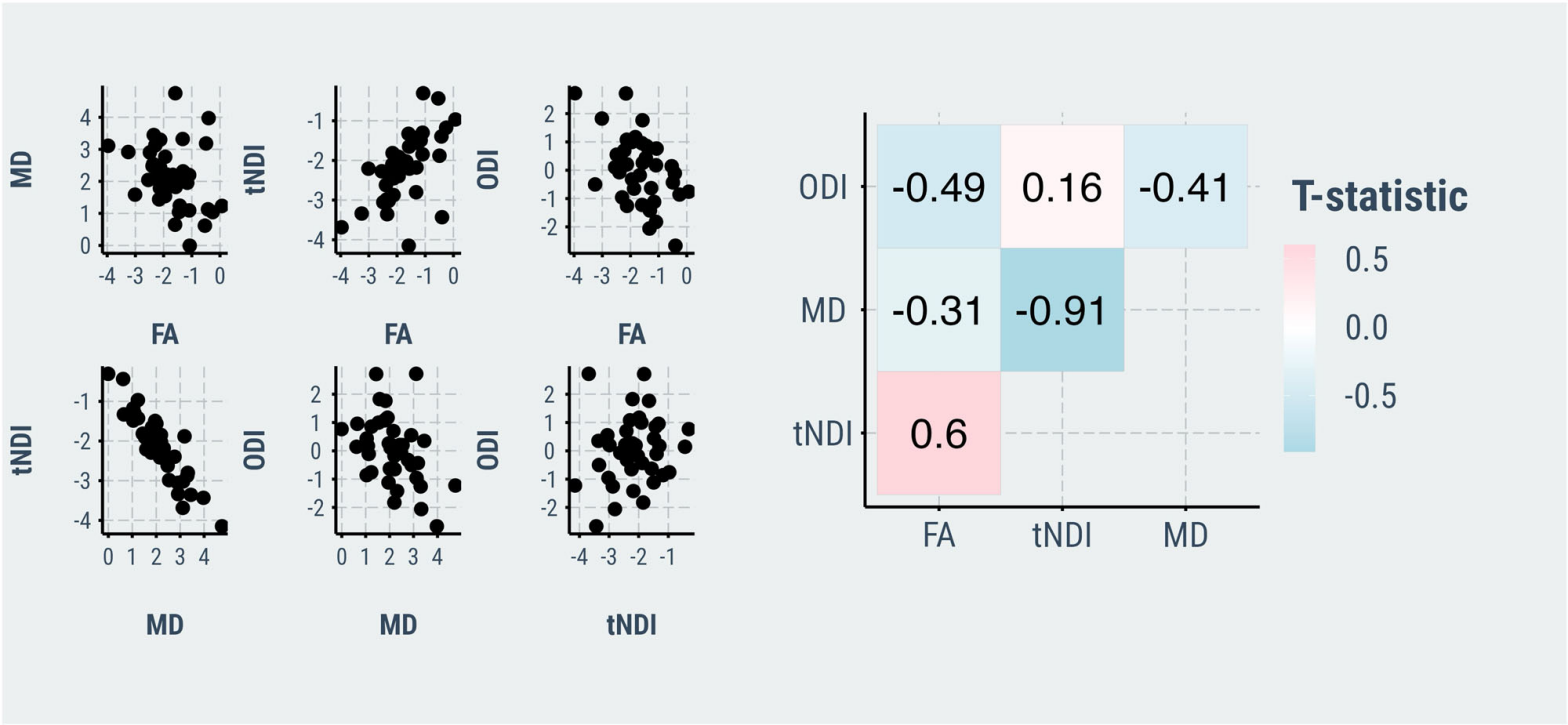
Scatter plots of T-statistics representing the directionality and magnitude of associations between cortical [18 F]-Flortaucipir SUVr with indices of white matter microstructure in DLBs, adjusting for age, APOE, and [11 C]-PiB SUVr. DLBs Dementia with Lewy bodies spectrum, FA Fractional anisotropy, MD Mean diffusivity; tNDI tissue-weighted Neurite Density Index SUVr Standardized uptake value ratios PiB Pittsburgh Compound B.

In our multivariable regression models adjusting for age, *APOE*, and [11 C]-PiB uptake, cortical [18 F]-Flortaucipir correlated with FA, MD, and tNDI in the DLBs group (Fig. 3). Forest plots for the region-specific regressions and corresponding T-statistics are provided in Supplementary Fig. 2. Specifically, greater cortical [18 F]-Flortaucipir SUVr was associated with lower FA across 2 white matter tracts, involving frontal lobes and limbic regions. Higher cortical [18 F]-Flortaucipir SUVr was associated with elevated MD in 12 tracts across the frontal, occipital, parietal, temporal lobes, and limbic regions. Higher cortical [18 F]-Flortaucipir SUVr was associated with lower tNDI in 11 tracts across the frontal, frontotemporal, occipital, frontal-occipital, temporal lobes, and limbic regions. No statistically significant associations were found between [18 F]-Flortaucipir SUVr and regional ODI in DLBs. Next, the Dice coefficients were calculated between the binarized sets of white matter tracts associated with [18 F]-Flortaucipir to assess the spatial similarity of tau associations among FA, MD, and tNDI. The resulting Dice coefficient between the topographies of tau-related MD and tNDI was 0.87, indicating a high degree of topological similarity between the two measures. Furthermore, the regional T-statistics of tau-related MD and tNDI were strongly, inversely correlated (*r* = −0.91), suggesting a similar magnitude in the strengths of the associations with regional MD and tNDI across all white matter tracts (Fig. 4). Elevated [11 C]-PiB SUVr was correlated with higher ODI values in the inferior temporal white matter white matter tract, after adjusting for age, *APOE*, and [18 F]-Flortaucipir. No other associations were found with [11 C]-PiB SUVr in relation to FA, MD, and tNDI values.

The application of the two-compartment PVC model did not significantly alter the correlations observed between regional DTI/NODDI parameters and [11 C]-PiB or [18 F]-Flortaucipir SUVr values. The topography of statistically significant correlations after correction for multiple comparisons was also broadly similar between non-PVC and PVC analyses, as detailed in Supplementary Fig. 3.

**Fig. 5:**
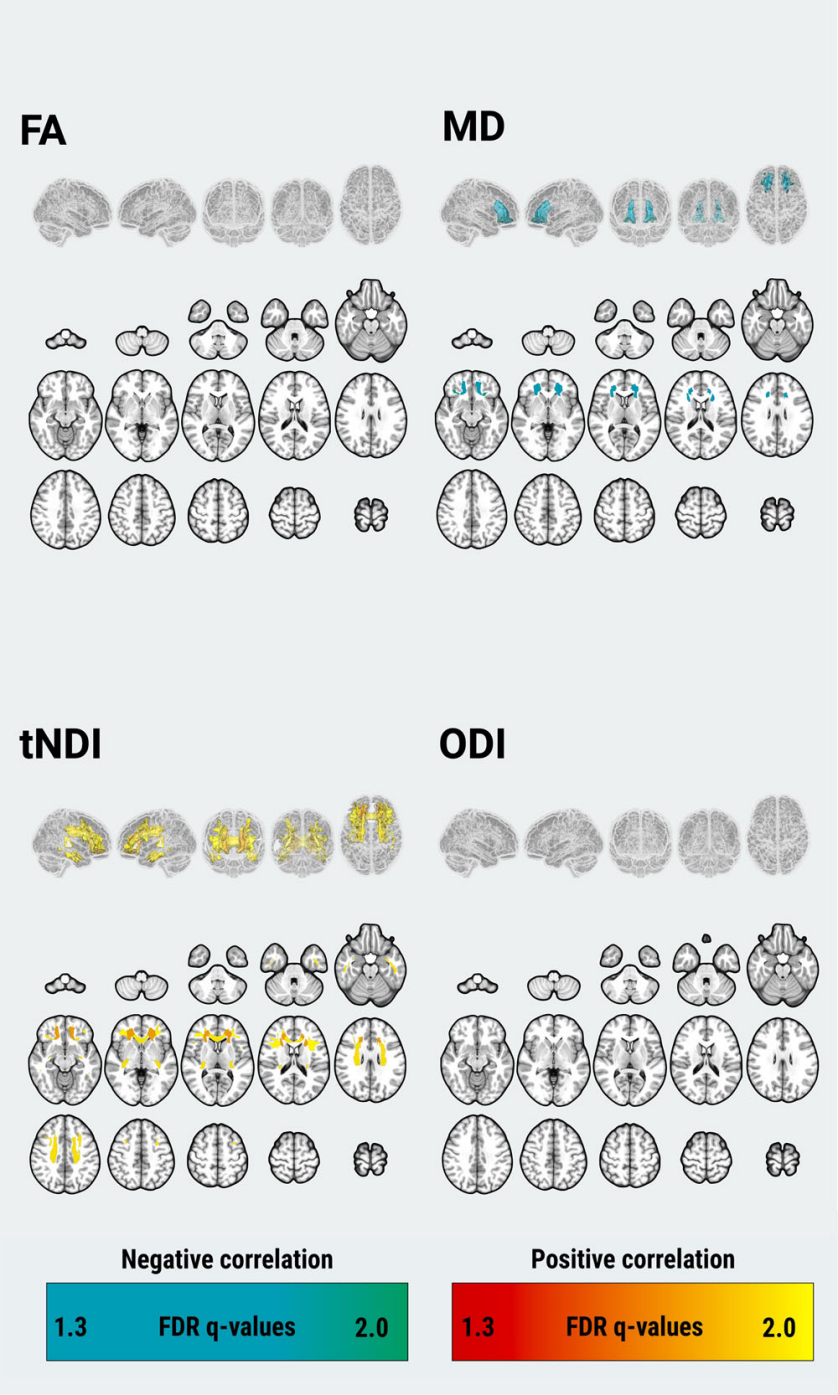
Relationship between tau and white matter microstructure in CU. Statistically significant associations of cortical [11 C]-PiB SUVr with regional DTI and NODDI parameters are depicted on 3D glass brain renderings and in volumetric MNI152 space (radiological orientation) for the CU controls. Linear regression models were adjusted for age, APOE genotype and [18 F]-Flortaucipir, and corrected for multiple comparisons with FDR. FDR q values are expressed as -log10(q) (i.e., 1.3 -> q = 0.05). Abbreviations: CU Cognitively unimpaired, DTI Diffusion tensor imaging, APOE Apolipoprotein E, FDR False Discovery Rate, FA Fractional anisotropy, MD Mean diffusivity, NODDI Neurite Orientation Dispersion and Density Imaging, tNDI tissue-weighted Neurite Density Index, ODI Orientation Dispersion Index, MNI152 Montreal Neurological Institute, SUVr Standardized uptake value ratios.

Among the matched CU controls, there were no associations of cortical [18 F]-Flortaucipir SUVr with DTI or NODDI parameters in any of the white matter tracts. However, higher cortical [11 C]-PiB SUVr was associated with lower MD in 2 white matter tracts, primarily in the frontal lobes (anterior corona radiata, lateral frontal-orbital white matter) after adjusting for age, *APOE*, [11 C]-Flortaucipir and corrected for multiple comparisons with FDR. Higher cortical [11 C]-PiB SUVr was also associated with elevated tNDI in 8 tracts across the frontal (anterior corona radiata, genu of the corpus callosum, inferior frontal white matter, lateral frontal-orbital white matter, and middle frontal white matter), frontoparietal (superior corona radiata) and temporal lobes (inferior temporal white matter and retro-lenticular internal capsule, Fig. 5). The corresponding forest plots of regional T-statistics are provided in Supplementary Fig. 4.

### Associations with clinical disease severity

**Fig. 6:**
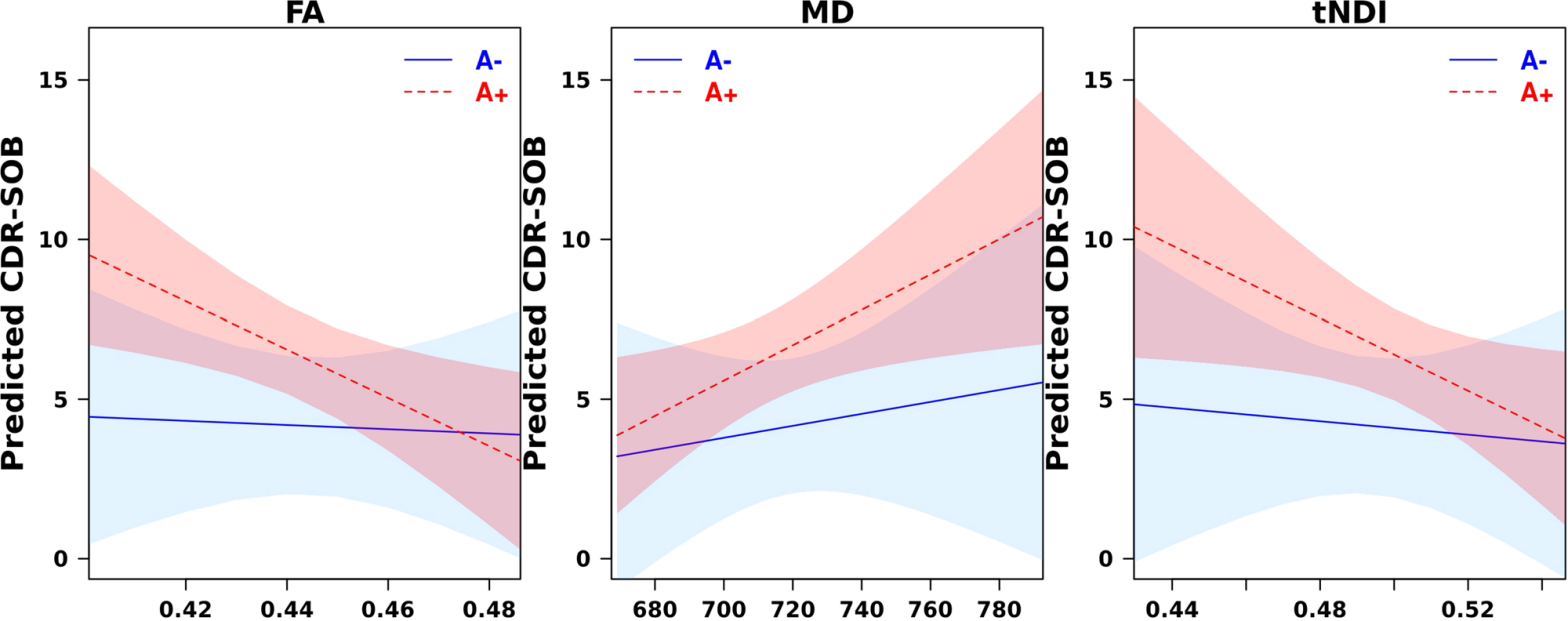
Associations between composite ROI measures of tau-associated FA, MD and tNDI with the CDR-SOB in the DLBs group. Amyloid-β status is denoted by color-coded lines with 95% confidence bands (blue = amyloid-β negative, red = amyloid-β positive). Tau-associated white matter injury was statistically significant with CDR-SOB exclusively in the subgroup of DLBs who are amyloid-β positive. A + amyloid-β positive DLBs, A- amyloid-β negative DLBs, CDR-SOB Clinical Dementia Rating Sum of Boxes, DLBs dementia with Lewy bodies spectrum, FA Fractional anisotropy, MD Mean Diffusivity; tNDI tissue-weighted Neurite Density Index, ROI Regions of Interest.

In the DLBs group, there were no statistically significant correlations between composite measures of tau-associated FA, MD, and tNDI with the CDR-SOB (FA: *r* = −0.28, *p* = 0.07; MD: *r* = 0.28, *p* = 0.07; tNDI: *r* = −0.22, *p* = 0.15, age-adjusted linear regressions). However, stratified analyses revealed statistically significant correlations in the subgroup of DLBs that were amyloid-β positive (FA: *r* = −0.51, *p* = 0.01; MD: *r* = 0.47, *p* = 0.02; tNDI: *r* = −0.43, *p* = 0.036, age-adjusted linear regressions). In contrast, these associations were not present in the amyloid-β negative DLBs (FA: *r* = −0.04, *p* = 0.86; MD: *r* = 0.13, *p* = 0.58; tNDI: *r* = −0.08, *p* = 0.75, age-adjusted linear regressions). There was one influential observation in the amyloid-β positive group which when removed weakened the correlations to insignificance with MD (*r* = 0.28, *p* = 0.20; age-adjusted linear regressions) and tNDI (*r* = −0.29, *p* = 0.18; age-adjusted linear regressions), but FA remained statistically significant (*r* = −0.42, *p* = 0.047; age-adjusted linear regressions). As a further sensitivity analysis, the use of robust linear regressions, in which all the data points were considered, confirmed that these associations remained statistically significant (FA: *p* = 0.024, MD: *p* = 0.026, and tNDI: *p* = 0.045; age-adjusted linear regressions). Lastly, we show the amyloid-βpositivity stratified linear regression analysis adjusting for age with the composite ROIs predicting CDR-SOB. The curves represent an average 70 year old in Fig. 6. The associated *p*-values are the same as the reported partial correlations.

### SEM of associations between AD biomarkers and white matter injury

**Fig. 7:**
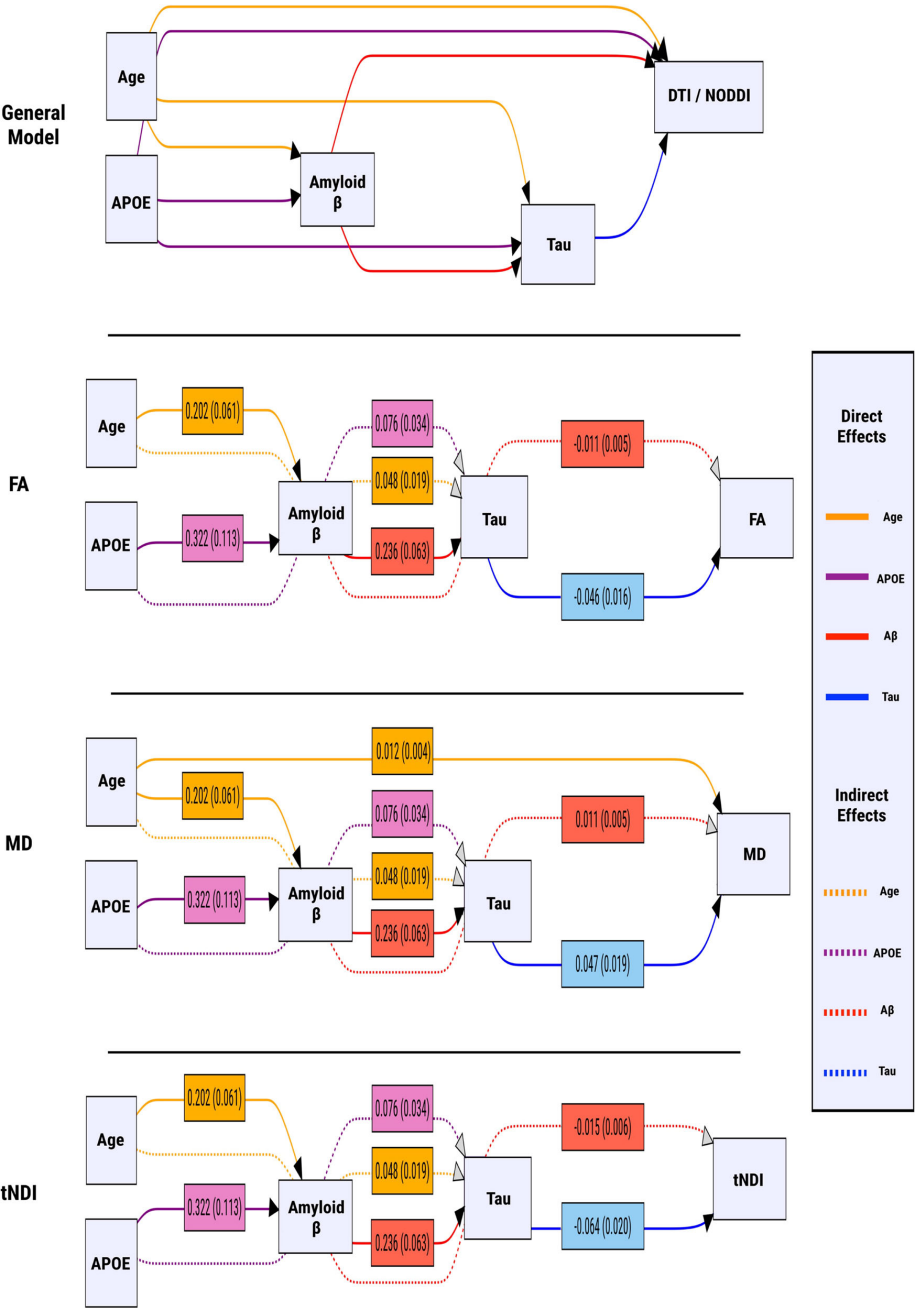
Relationships among age, APOE, cortical amyloid-β, tau PET biomarkers, and white matter microstructural injury in DLBs were examined with SEMs. All direct and indirect effects visualized in this figure are statistically significant (*p* < 0.05). Age and APOE genotype were included as modifiers of all variables in DLBs. Arrows that join nodes indicate direct effects, while arrows that join nodes after passing through intervening mediator variables indicate indirect effects. Total effects are the sum of direct and indirect effects. The color-coding of both direct and indirect effects reflects the predictors. The effects are summarized using regression coefficients and associated standard errors. APOE Apolipoprotein E, DLBs Dementia with Lewy bodies spectrum, DTI Diffusion tensor imaging; NODDI Neurite Orientation Dispersion and Density Imaging, PiB Pittsburgh compound B, SUVr Standardized uptake value ratios, SEM Structural equation model, F Fractional anisotropy, MD Mean Diffusivity, tNDI tissue-weighted Neurite Density Index.

Median values of DTI and NODDI parameters were extracted from the composite white matter ROIs that exhibited statistically significant pairwise differences between DLBs and CU controls after FDR correction for multiple comparisons. Subsequently, path analyses using SEMs were conducted to explore the multivariable associations among age, APOE genotype, cortical amyloid-β, cortical tau, and white matter injury. All SEM models demonstrated acceptable goodness of fit. SEMs estimate direct effects (joining two variables), indirect effects (joining two variables but passing through intervening mediator variables), and total effects (the sum of the direct and indirect effects). The effects obtained from the analysis are depicted in Fig. 7 and tabulated in Supplementary Table 2. Both age and *APOE* genotype had statistically significant direct effects on amyloid-β. A positive direct effect of amyloid-β on tau was also observed, while age and *APOE* genotype exerted statistically significant indirect effects on tau levels through their influence on amyloid-β. In terms of white matter microstructural injury, cortical amyloid-β showed no significant direct effects on FA, MD, and tNDI, although it exerted statistically significant indirect effects on lower FA, elevated MD, and lower tNDI through its influence on tau. Cortical tau, on the other hand, had statistically significant direct effects on lower FA, higher MD, and lower tNDI. Furthermore, age had a statistically significant total effect on higher MD.

## Discussion

Despite the growing body of evidence from DTI studies showing widespread white matter abnormalities in DLB, inherent limitations of the DTI model have hindered definitive conclusions about the biological mechanisms underlying deficits in FA and MD. Furthermore, the influence of co-existing amyloid-β and tau on white matter integrity is still unclear in Lewy body diseases. Here, we addressed both issues by integrating multi-shell NODDI with [11 C]-PiB and [18 F]-Flortaucipir PET imaging in a well-characterized group of individuals on the DLB spectrum. Firstly, we confirmed previously reported white matter injury in terms of lower FA and higher MD in DLBs compared to similarly matched cognitively unimpaired individuals^17,18^ and provided further biological specificity by demonstrating that the patterns of lower FA and higher MD mapped onto regions with lower tNDI values, indicative of axonal loss in white matter tracts. Secondly, we found that greater cortical [18 F]-Flortaucipir uptake was associated with a stereotypical AD pattern of white matter injury that was, in turn, associated with worse clinical disease severity, particularly in the presence of elevated amyloid-β deposition. Finally, our findings were substantiated by SEM analyses, which revealed nuanced associations of amyloid-β on white matter injury through its influence on tau deposition.

The spatial extent and magnitude of white matter abnormalities in DLB have been extensively studied using DTI^17–19^, however, the underlying pathological mechanisms have thus far remained elusive due to the inherent lack of specificity of the DTI model. For instance, reduced neurite density and increased dispersion of fiber orientations could both lead to a reduction in FA, while MD may be influenced by a multitude of processes such as edema, demyelination, and axon loss. Here, our study revealed a widespread decline in microstructural integrity, marked by lower FA and elevated MD spanning across major white matter tracts in the frontal, occipital, parietal, temporal, and limbic regions. Building upon previous research, our application of NODDI demonstrated a substantial overlap between elevated MD and reduced tNDI in DLBs. Notwithstanding the need for further histological validation of NODDI parameters^29^, these findings suggest that the widespread extent of lower FA and elevated MD is more likely underpinned by reduced axonal density as opposed to increased dispersion of fiber orientations or axonal fanning. Indeed, the accumulation of misfolded α-synuclein has been shown to disrupt axonal transport mechanisms that are essential for maintaining neuronal function and survival, ultimately leading to axonal degeneration and loss^30^.

Previous studies have demonstrated that cortical tau deposition is associated with white matter injury in AD dementia^31^ and pathologically diagnosed AD cases^20^. The present study replicated these findings and extended this relationship to a well-characterized group of individuals on the DLB spectrum. Higher [18 F]-Flortaucipir SUVr was associated with focal reductions in FA and broader patterns of elevated MD and reduced tNDI primarily involving the limbic and inferior temporal white matter regions, consistent with the loss of microtubule stability from tau hyperphosphorylation, leading to axonal transport disruptions and impaired axonal integrity^20^. Notably, these associations persisted even after accounting for age, *APOE* genotype, and cortical amyloid-β deposition.

In comparison to FA, additional tracts where tau was associated with elevated MD and reduced tNDI involved the ventral limbic projections and inferior temporal white matter projections, all of which are known to have dense projections within the “cortical neurodegeneration signature” of AD^32^, a select group of regions that are susceptible to early neurofibrillary tangle accrual in AD^33–35^. In this regard, our previous finding that tau PET was longitudinally associated with atrophy rates in cortical regions that are proximal to the tau-related white matter tracts herein (i.e, hippocampus and temporoparietal cortices) raised the intriguing possibility that the spread of tau pathology from the affected white matter tracts to the adjacent gray matter regions may lead to cell death and subsequent atrophy as seen on MRI^36^. Interestingly, there were also sparse associations of tau deposition with white matter injury that were not confined to the limbic or inferior temporal tracts, such as the distal white matter tracts (i.e., inferior frontal white matter) extending towards the occipital regions. Notwithstanding the cross-sectional design, one could cautiously speculate that tau deposition may have distal effects on white matter integrity, potentially affecting cognitive function beyond the limbic system in DLBs. Future research should aim to elucidate the mechanisms by which tau contributes to white matter injury across various brain regions and to determine the consequent effects on cognitive function.

The spatial distribution of white matter abnormalities in DLBs (Fig. 2) exceeded regions associated with tau pathology (Fig. 3). This observation suggests that the widespread pattern of white matter deficits in DLBs cannot be solely attributed to concomitant AD pathologies. α-synuclein is likely an independent driver and possibly works synergistically with amyloid-β, tau, and neuroinflammation. As more specific α-synuclein biomarkers emerge^37^, examining their relationships to multi-modal signatures of white matter injury will help delineate the relative contributions of accumulating amyloid-β, tau, and neuroinflammation^38^.

Statistically significant correlations were observed between elevated MD and reduced FA and tNDI values in tau-related white matter tracts, and greater clinical disease severity in DLBs, especially in individuals positive for amyloid-β. Interestingly, these findings may not be exclusive to DLB, as our group has previously reported similar results in post-mortem AD cases, where higher MD and lower FA were associated with both greater NFT burden and worse CDR-SOB in spatially overlapping white matter regions^20^. The consistency of findings, despite differences in methodologies (i.e., in vivo [18 F]-Flortaucipir PET *versus* post-mortem NFT burden) and neurodegenerative disease groups (i.e., DLBs *versus* AD), reinforced the robustness of our findings and collectively implicated tau-related white matter damage as a significant driver of disease course in people with DLBs. More broadly, these observations also highlighted the potential of DTI and NODDI techniques as surrogate markers of early tau-related injury in white matter microstructure. For example, the limbic patterns of white matter meta-ROIs may serve as novel diffusion MRI biomarkers to aid in earlier detection and monitoring of disease progression, or potentially as an outcome measure to evaluate the efficacy of new interventions targeted towards the preservation of axonal integrity.

In this study, [11 C]-PiB SUVr did not exhibit significant direct associations with DTI or NODDI parameters in DLBs. This is broadly in keeping with post-mortem data suggesting a negligible impact of neuritic plaques on DTI deficits in AD^20^. Nonetheless, the significant indirect effects of [11 C]-PiB SUVr on reduced FA, increased MD, and lower tNDI from our SEMs suggest that the relationship between amyloid-β and white matter deficits is more intricate than previously believed, potentially affecting white matter indirectly through its impact on downstream tau pathology. Indeed, this notion is aligned with the “amyloid-β hypothesis”^39^, which posits that amyloid-β aggregation initiates a cascade of events eventually leading to the formation of NFT, which is more strongly associated with white matter deficits^20,21^ and gray matter atrophy^35,40^. The general pathway of amyloid-β → NFT → white matter injury is also aligned with histopathological evidence of synergistic interactions between α-synuclein, amyloid-β, and tau in DLB. Similarly, cortical amyloid-β load has been found to strengthen the association between tau and functional connectivity impairments^41^, as well as gray matter atrophy^42^. In summary, though not directly linked to white matter integrity, our findings suggest that upstream amyloid-β may propagate the deleterious downstream effects on structural connectivity via its influence on tau, and therefore underscore the therapeutic potential for early anti-amyloid-β interventions to mitigate the downstream consequences mediated through tau^43^.

Although our study was not designed to conduct a methodological comparison between DTI and NODDI, we were able to assess the relative sensitivity of both techniques to microstructural injury in DLBs by evaluating spatial patterns of group differences in FA, MD, and tNDI. Interestingly, substantial overlap was observed, without clear evidence of tNDI conferring additional sensitivity over conventional DTI metrics. One plausible explanation is that tNDI may be more sensitive during the early stages of neurodegenerative diseases, during which axonal degeneration is particularly salient^44^. In contrast, overall water mobility changes measured by MD may only become more apparent with substantial tissue damage, demyelination, and neuronal death have taken place. Since there were relatively few MCI-LB patients in the DLBs group (*n* = 13), we have refrained from exploring the potential early-stage white matter involvement in this cohort. Future longitudinal studies comparing both imaging modalities across different stages of DLB could help determine when both techniques are most sensitive to white matter microstructural injury. Furthermore, as suggested in previous research, the higher sensitivity to group differences in conventional DTI markers may also be due to the fewer model assumptions in DTI compared to NODDI, such as the absence of a fixed value for intrinsic diffusivity or the lack of separate diffusion compartments in the DTI model^45^.

The absence of statistically significant correlations between tau and any of the DTI or NODDI metrics in the CU group in support of the notion that tau-related white matter injury in DLBs may be driven by factors intrinsic to Lewy body pathology, such as the interplay between α-synuclein and tau, and only at higher levels of tau accumulation that exceed the [18 F]-Flortaucipir SUVr levels observed in the CU group^2^. The absence of tau pathology in the CU group could have diminished the statistical strength to detect notable connections with DTI and NODDI parameters. Interestingly, we observed an association between elevated amyloid-β levels and lower MD as well as higher tNDI in the frontal white matter tracts of CU individuals. Although this finding may appear to challenge the conventional understanding that AD biomarkers are intimately associated with impaired microstructural integrity, there have been several exceptions to this trend, such as higher FA in amyloid-β-positive CU individuals^46^ and non-linear associations between [11 C]-PiB and DTI parameters, where amyloid-β was correlated with preserved white matter microstructure at low levels^47,48^. Despite the modest sample size of CU participants in our study and the higher frequency of men due to sex-matching, a plausible explanation for the counter-intuitive pattern of correlations could be related to the concept of brain reserve. Such paradoxical interactions may reflect compensatory processes in resilient individuals who can tolerate the incipient accrual of AD pathologies without exhibiting clinical decline. Exploring this hypothesis is beyond the scope of the present study, and further research with larger, diverse cohorts of CU individuals would be ideal to elucidate the role of brain resilience in mitigating the onset of clinical symptoms.

Several caveats should be considered when interpreting our findings. (a) Although we did not have autopsy confirmation in this study, the uncertainty of antemortem diagnosis is mitigated by the high autopsy confirmation rates of clinical diagnosis at our center (89%). (b) Our cohort was predominantly composed of males, which may not fully represent the disease course in female patients with DLBs. This is unlikely to have led to false positives since previous research has indicated that women with DLBs may have a more aggressive disease course, severity of cognitive impairment, and higher rates of AD biomarker positivity^49,50^. (c) Although our SEMs were designed with biological plausibility in mind, longitudinal studies are still needed to establish NODDI change rates and how they may relate to clinical decline, longitudinal accumulation of tau pathology^36^, and amyloid-β over time^51^. (d) Although our study included individuals with prodromal DLBs, the statistical analyses were not sufficiently powered to discern group differences at the MCI-LB stage. Ongoing data collection and future research should delineate the patterns of NODDI deficits in prodromal DLB and investigate whether coexisting AD may exacerbate the transition from MCI-LB to probable DLB. Despite these limitations, our study aimed to investigate microstructural injury across the entire DLB spectrum by including both prodromal and established DLB cases. Additionally, it is worth noting that among the 13 individuals with MCI-LBs, 7 individuals were subsequently diagnosed with probable DLB at follow-up clinical assessments, further supporting our rationale for combining MCI-LB with probable DLB. (e) While our study characterized the correlations of AD co-pathology with white matter injury in DLBs, the complex interactions among amyloid-β, tau, and alpha-synuclein – each of which may promote downstream aggregation of the others^15^— on DTI and NODDI abnormalities remain unclear. An important future direction is to leverage larger prospective, longitudinal cohorts to allow stratification of DLB participants based on amyloid-β and tau status. Comparing NODDI parameters in subgroups not only to each other but also healthy controls would help disentangle additive versus synergistic interactions between AD and α-synuclein pathologies on white matter injury and clinical decline.

In this original research, we integrated biophysical NODDI parameters with multi-modal PET imaging of amyloid-β and tau. Our findings suggest that axonal injury is a significant pathological factor contributing to widespread microstructural deficits in DLBs. Furthermore, our study expands the literature on tau-related white matter deficits in AD^20,21^ by revealing a similar relationship in Lewy body diseases. Considered alongside the growing body of evidence for tau-related cognitive decline and gray matter atrophy in DLB^36,52^, these findings further implicate tau pathology as a potent disease modifier in DLB and may have potential implications for disease-modifying trials that aim to target not only Lewy body pathology but also AD in individuals with DLB.

## Methods

### Selection of participants

We included consecutive patients with clinically probable DLB^53^ who were at mild to moderate clinical stages (*n* = 32) and those with prodromal DLB (*n* = 13)^54^ to comprise a group of people on the DLB spectrum (*n* = 45) who were enrolled at the Mayo Clinic Alzheimer’s Disease Research Center between February 2018 and October 2021. Clinical disease severity was measured with the Clinical Dementia Rating – Sum of Boxes (CDR-SOB), and cognitive performance was evaluated with the Mini-Mental State Examination (MMSE)^55^ We also assessed clinical features characteristic of DLB: (1) Unified Parkinson Disease Rating Scale, part III (UPDRS-III) for parkinsonism; (2) visual hallucinations characterized by being fully formed, not restricted to a single episode and not related to another medical issue; (3) cognitive fluctuations defined as a score of 3 or 4 on the Mayo Fluctuations Questionnaire^56^; and (4) probable REM sleep behavior disorder (RBD) based on the International Classification of Sleep Disorders-II diagnostic criteria^57^. Clinical diagnosis was established by a consensus committee including behavioral neurologists, neuropsychologists, and study coordinators. An automated greedy match algorithm was used to match 1:1 the CU controls (*n* = 45) on age and gender from the Mayo Clinic Study of Aging, which is an epidemiologic study of aging in Olmsted County, MN^58^. All participants in the study met specific inclusion criteria, including having multi-shell diffusion data, amyloid-β, and tau PET scans. Exclusion criteria for the DLBs were a history of traumatic brain injury, hydrocephalus or intracranial mass, a history of chemotherapy, head radiation therapy, or substance abuse, and having neurologic or psychiatric disorders other than DLB. The Mayo Clinic Institutional Review Board approved the study. Informed consent for participation was obtained from all patients or a surrogate according to the Declaration of Helsinki.

### Neuroimaging

All participants underwent a brain MRI protocol on one of three identical Siemens Prisma 3 T scanners running VE11 software (uniform versions) equipped with 64-channel receiver head coils. The acquisition protocols were consistent across all three scanners: a magnetization-prepared rapid gradient echo (MPRAGE) sequence with a resolution of 0.8 mm isotropic and a diffusion scan using VE11 Simultaneous Multi-Slice acceleration with adaptive coil combination. The field of view was 232 mm in X and Y and 162 mm in the Z direction with 2.0 mm isotropic voxels for the diffusion scan. The data consisted of 127 volumes, including 13 non-diffusion-weighted images and 114 diffusion-encoding gradient directions. The diffusion-weighted images were evenly spread over the entire spherical shells using an electrostatic repulsion model and were interspersed in time to minimize gradient heating^59^. The echo time and repetition time were 71 ms and 3400 ms, respectively. The diffusion scan included b-values of 0, 500, 1000, and 2000 s/mm^2^.

PET imaging was performed on Siemens and General Electric PET/CT scanners. The 11C-PiB scans comprised four 5 min dynamic frames captured from 40 to 60 min post-injection (average 596 MBq; range 292–729 MBq). For Flortaucipir PET scans, an intravenous bolus injection of an average of 370 MBq (range 333–407 MBq) was administered, followed by an 80 min uptake period. Subsequently, a 20 min scan consisting of four 5-min frames was obtained. All PET images underwent visual inspection for technical quality. Detailed information on PET data preprocessing can be found in our previous studies^36,60,61^.

### Neurite orientation dispersion and density imaging

We used an in-house pipeline to preprocess the diffusion datasets, which were visually inspected by trained analysts. An intracranial mask was created for the diffusion MRI scan^62^ and noise was estimated and removed using random matrix theory^63^. Head motion and eddy current distortion were corrected using FSL’s eddy_cuda, followed by correction of Gibbs ringing^64^ and Rician bias^65^. Diffusion tensors were fitted using a non-linear least-squares fitting algorithm implemented in DIPY to generate FA and MD images for both the multi-shell and extracted *b* = 1000 data. The NODDI model was fitted using the Accelerated Microstructure Imaging via Convex Optimization (AMICO) implementation in Python^66^, producing voxelwise maps of Orientation Dispersion Index (ODI), Neurite Density Index (NDI), and free water (IsoVF). We calculated a tissue-weighted NDI (tNDI) by adjusting the original NDI measurement with a scaling factor based on (1-IsoVF), which accounts for the presence of free water, cerebrospinal fluid, and other extracellular spaces. This allowed us to obtain a more precise measurement of neurite density, henceforth referred to as tNDI. To generate regional measures of FA, MD, tNDI, and ODI for each bilateral white matter tract, we first registered the JHU ‘Eve’ WM atlas^67^ to participant native space using Advanced Normalization Tools – Symmetric Normalization (https://stnava.github.io/ANTs/)^68^. Next, bilateral median values of FA, MD tNDI, and ODI were computed, weighted by the size of each region of interest (ROI). A full list of white matter parcellations is provided in Supplementary Table 1.

### PET imaging of amyloid-β and tau

PET images were analyzed with our in-house fully automated image processing pipeline as previously described^69^. Each tau PET image was rigidly registered to its corresponding MPRAGE using SPM12^70^ and regional PET values were extracted from automatically labeled 38 ROIs propagated from the Mayo Clinic Adult Lifespan template (MCALT, https://www.nitrc.org/projects/mcalt/)^71^. For each participant scan, we calculated a composite cortical amyloid-β standardized uptake value ratio (SUVr) by taking the median uptake in six ROIs: prefrontal, orbitofrontal, parietal, temporal, anterior cingulate, and posterior cingulate/precuneus, and normalizing by the median amyloid-β PET uptake in the cerebellar crus gray matter. Similarly, we computed a composite [18 F]-Flortaucipir SUVr by taking the median tau PET uptake in six ROIs: entorhinal, amygdala, parahippocampal, fusiform, inferior temporal, and middle temporal, and normalizing by the median tau PET uptake in the cerebellar crus gray matter for each participant. These composite SUVr values provided cortical summary measures of amyloid-β and tau uptake in the brain, which we then used as predictor variables to investigate associations with regional DTI and NODDI parameters.

### Statistical analyses

The baseline demographic, clinical, and cognitive characteristics were summarized using means and standard deviations for continuous variables and counts and percentages for categorical variables. We used conditional logistic regression models to compare group differences in DTI and NODDI parameters, accounting for the matching. All amyloid-β and tau SUVRs were analyzed with log transformations due to skewness. Dice coefficient indices were computed between the binary maps representing the FDR-corrected group differences in terms of FA, MD, and tNDI between DLBs and CU groups. The formula for the Dice coefficient is shown in Eq. (1): where A and B are the two binary maps.

1 $${\rm{Dice}}\,{\rm{Coefficient}}=\frac{2\times \left|A\cap B\right|}{\left|A\right|+\left|B\right|}$$

To investigate the influence of amyloid-β and tau on regional DTI and NODDI parameters, separate multivariable regression models were constructed in the DLBs and CU groups while adjusting for age and APOE genotype and correcting for multiple comparisons across white matter ROIs using False Discovery Rate (FDR).

In addition to our primary analyses, we conducted supplementary analyses using the two-compartment model to obtain partial volume corrected (PVC) SUVr from the [11 C]-PiB and [18 F]-Flortaucipir PET scans^72^.

Subsequently, we derived composite ROIs based on the statistically significant FDR results representing white matter injury associated with tau and evaluated both age-adjusted partial Pearson’s correlations and age-adjusted linear regressions between these composite ROIs and the CDR-SOB in the DLB group. We used residual analyses to evaluate the validity of regression assumptions. Because we had an influential observation in a couple of models we ran the models with and without the observation and also ran robust regressions with Huber estimators as sensitivity analyses.

To further explore the relationships among age, APOE, amyloid-β, tau, and white matter injury, path analyses (SEMs with only manifest variables) were employed using Mplus version 8.10. The SEM models were designed such that variables on the left were considered upstream of variables on the right. Direct effects, indirect effects, and total effects were analyzed to investigate potential associations among the variables. Age and *APOE* genotype were included in the model as exogenous predictors of AD biomarkers and white matter injury in DLBs, which were represented by composite ROIs obtained from statistically significant pairwise group differences between the DLBs and CU groups (FDR *q* < 0.05). Finally, path analyses were pruned using multiple goodness of fit measures, including the Root Mean Square Error of Approximation (RMSEA), the Standardized Root Mean Square Residual (SRMR), the Tucker-Lewis Index (TLI), the Comparative Fit Index (CFI), and *p*-values.

### Reporting summary

Further information on research design is available in the Nature Research Reporting Summary linked to this article.

### Supplementary information

Supplementary Materials

Reporting Summary

## Data Availability

Anonymized data will be shared by request from a qualified investigator in accordance with the Mayo ADRC data sharing protocol. A Data Use Agreement is required for access, which outlines the terms and conditions for secure data storage, protection, and compliance with data protection regulations and ethical guidelines. Requests for the data should be directed to the corresponding author, Professor Kejal Kantarci (kantarci.Kejal@mayo.edu).

## Code availability

The code used to derive the principal findings of this study are available upon request.
