# Supplementary material for: Influences of amyloid-β and tau on white matter neurite alterations in dementia with Lewy bodies
Source: NPJ Parkinsons Dis. 2024 Apr 3;10:76. doi: 10.1038/s41531-024-00684-4 (PMC10991290; doi:10.1038/s41531-024-00684-4)
Supplement: Supplementary file 1 — Supplementary Materials [file 41531_2024_684_MOESM1_ESM.pdf]

SUPPLEMENTARY MATERIALS

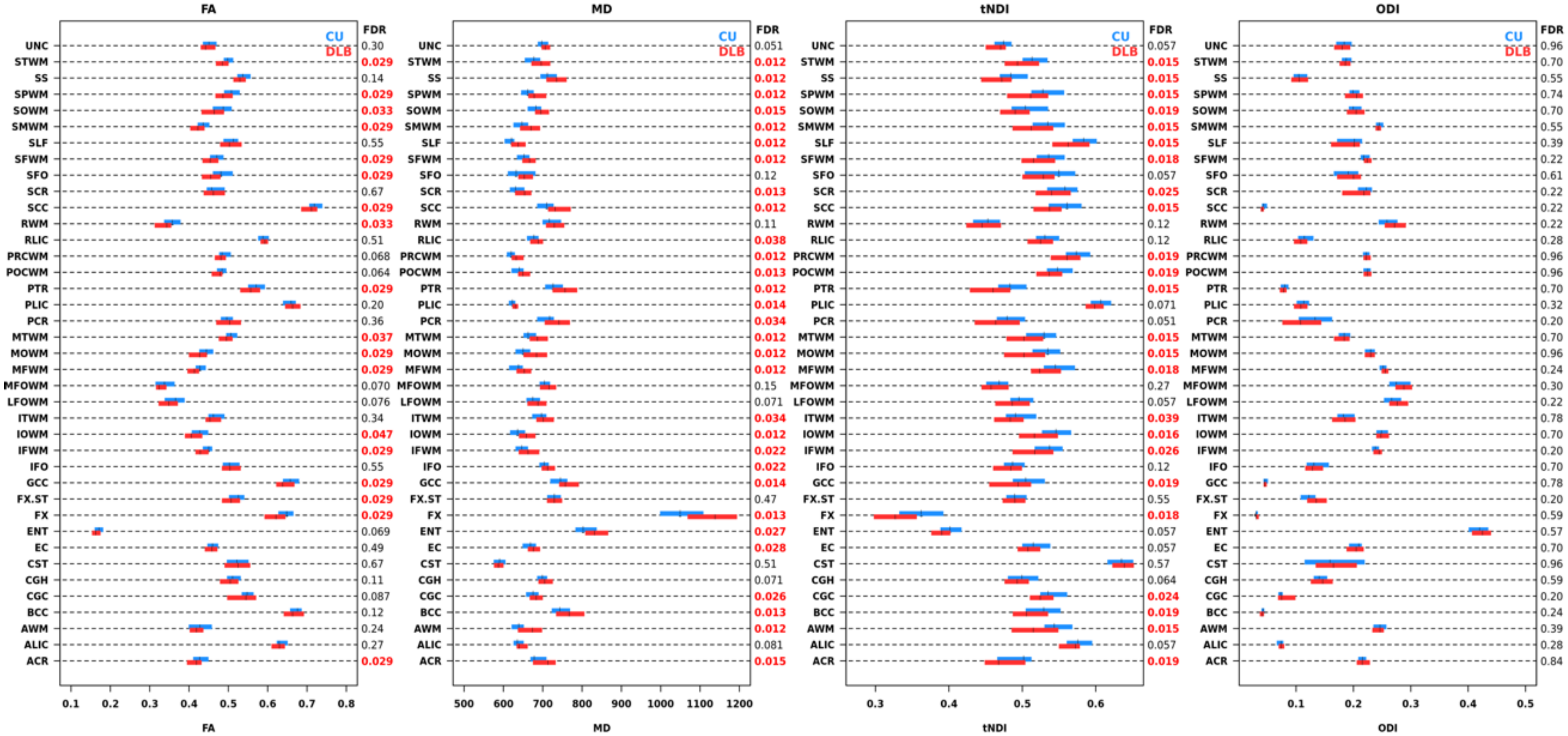

Supplementary Figure 1. Forest plots depicting regional pairwise group comparisons from conditional logistic models between DLBs and CU. Statistically significant group differences are marked in red (FDR $q < 0.05$). Abbreviations: DLBs = Dementia with Lewy bodies spectrum; CU = Cognitively unimpaired; FA = Fractional anisotropy; MD = Mean diffusivity; tNDI = tissue-weighted Neurite Density Index; ODI = Orientation Dispersion Index; FDR = False Discovery Rate.

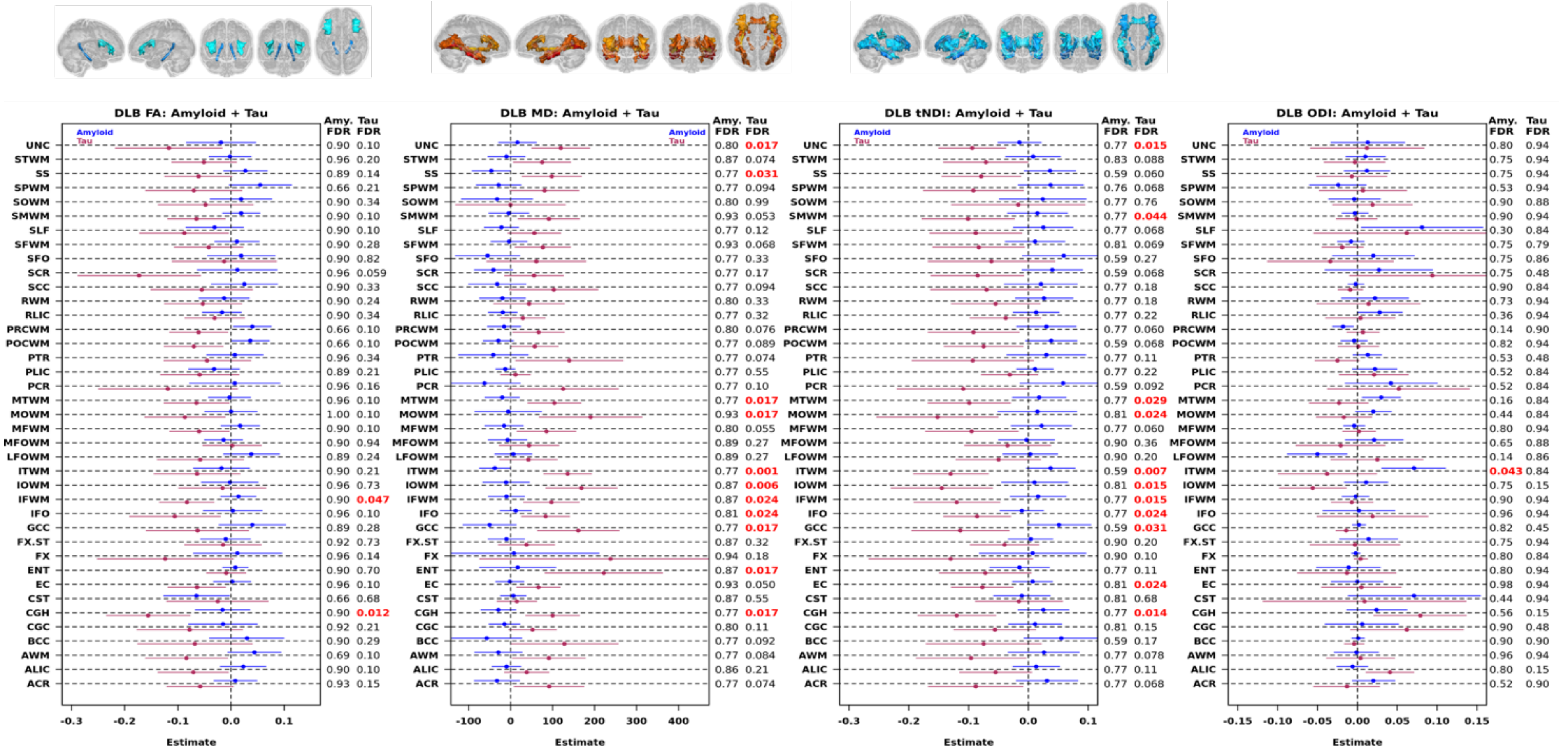

Supplementary Figure 2. Forest plots depicting the regional associations of [11C]-PiB SUVr and [18F]-Flortaucipir SUVr with DTI and NODDI deficits in DLBs. Dashed lines show 95% confidence intervals. Statistically significant results are marked in red (FDR q < 0.05). Abbreviations: DLBs = Dementia with Lewy bodies spectrum; DTI = Diffusion tensor imaging; NODDI = Neurite Orientation Dispersion and Density Imaging; PiB = Pittsburgh compound B; SUVr = Standardized uptake value ratios; FA = Fractional anisotropy; MD = Mean diffusivity; tNDI = tissue-weighted Neurite Density Index; ODI = Orientation Dispersion Index; FDR = False Discovery Rate.

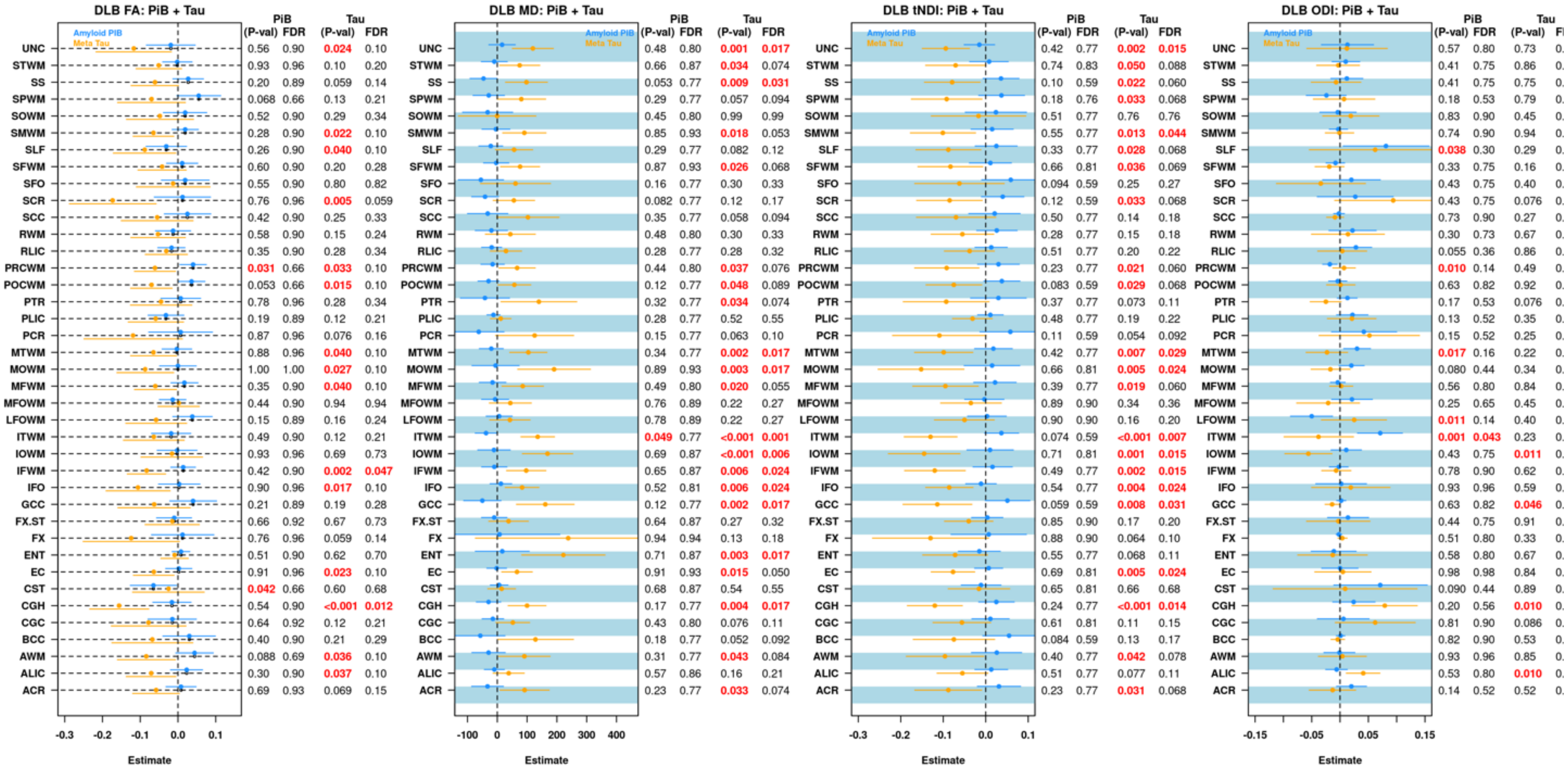

Supplementary Figure 3. Forest plots depicting the regional associations of PVC-ed [11C]-PiB SUVr and [18F]-Flortaucipir SUVr with DTI and NODDI deficits in DLBs. Dashed lines show 95% confidence intervals. Statistically significant results (FDR q < 0.05) are marked in red. Abbreviations: DLBs = Dementia with Lewy bodies spectrum; DTI = Diffusion tensor imaging; NODDI = Neurite Orientation Dispersion and Density Imaging; PiB = Pittsburgh compound B; SUVr = Standardized uptake value ratios; FA = Fractional anisotropy; MD = Mean diffusivity; tNDI = tissue-weighted Neurite Density Index; ODI = Orientation Dispersion Index; FDR = False Discovery Rate; PVC = Partial volume corrected using the two-Compartment model.

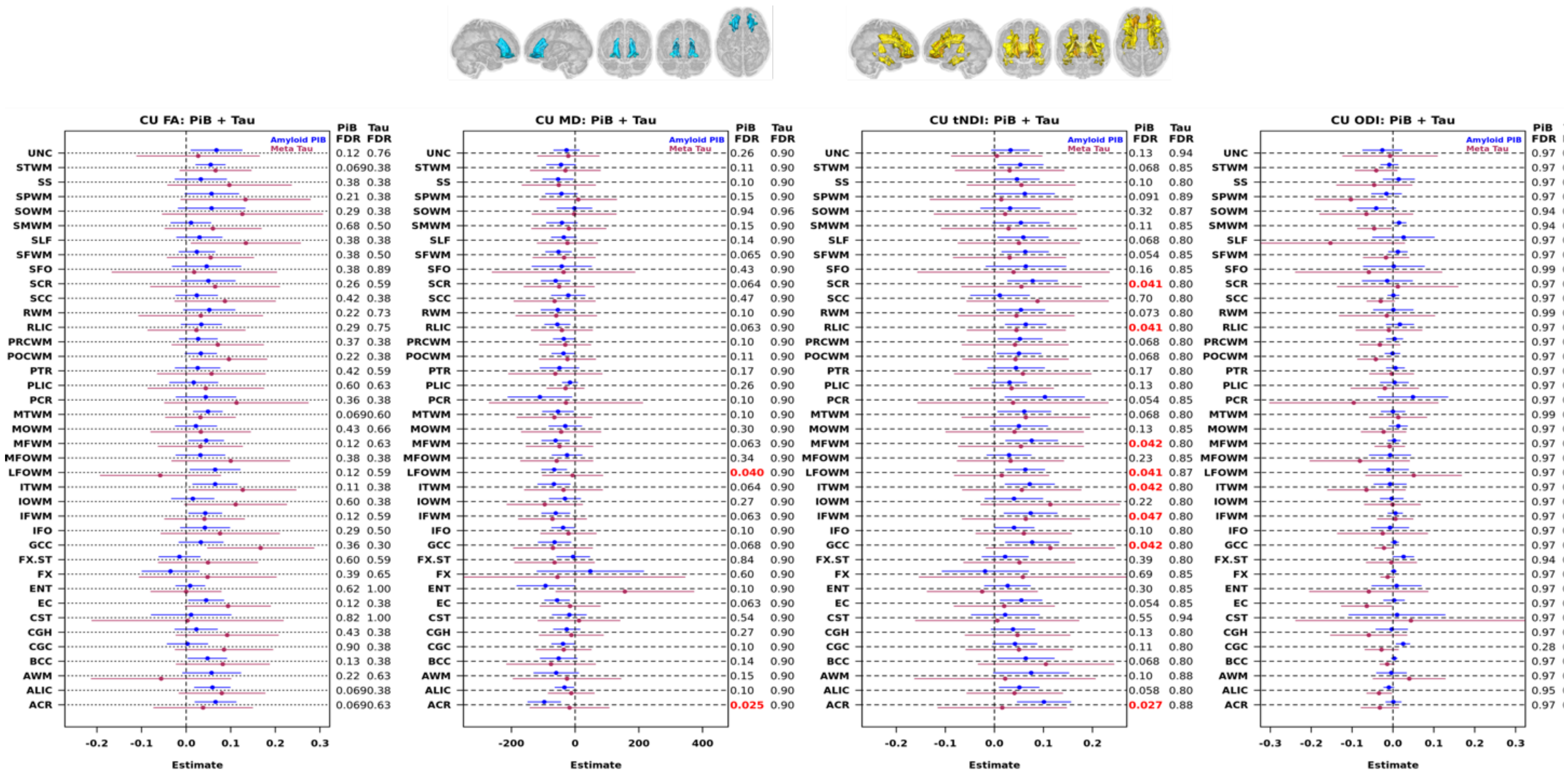

Supplementary Figure 4. Forest plots depicting the regional associations of [11C]-PiB SUVr and [18F]-Flortaucipir SUVr with DTI and NODDI deficits in the CU group. Dashed lines show 95% confidence intervals. Statistically significant results are marked in red (FDR q < 0.05). Abbreviations: CU = Cognitively unimpaired; DTI = Diffusion tensor imaging; NODDI = Neurite Orientation Dispersion and Density Imaging; PiB = Pittsburgh compound B; SUVr = Standardized uptake value ratios; FA = Fractional anisotropy; MD = Mean diffusivity; tNDI = tissue-weighted Neurite Density Index; ODI = Orientation Dispersion Index; FDR = False Discovery Rate.

Abbreviated Labels	Full Names of ROIs	Abbreviated Labels	Full Names of ROIs
ACR	Anterior Corona Radiata	SFO	Superior Frontal Occipital Fasciculus
ALIC	Anterior Limb of Internal Capsule	SFWM	Superior Frontal White Matter
AWM	Angular White Matter	SLF	Superior Longitudinal Fasciculus
BC	Body of Corpus Callosum	SMWM	Supramarginal White Matter
CGC	Cingulate Gyrus	SOWM	Superior Occipital White Matter
CGH	Cingulum Bundle (Hippocampal)	SPWM	Superior Parietal White Matter
CST	Corticospinal Tract	SS	Sagittal Stratum
EC	External Capsule	STWM	Superior Temporal White Matter
ENT	Entorhinal White Matter Area	UNC	Uncinate Fasciculus
FX	Fornix Column and Body		
FXST	Fornix Cresent and Stria Terminalis		
GCC	Genu of Corpus Callosum		
IFO	Inferior Fronto-Occipital Fasciculus		
IFWM	Inferior Frontal White Matter		
IOWM	Inferior Occipital White Matter		
ITWM	Inferior Temporal White Matter		
IFOWM	Lateral Frontal Orbital White Matter		
MFOWM	Middle Frontal Orbital White Matter		

Supplementary Table 1. List of white matter ROIs. Abbreviations: ROI = Regions of Interest.

Parameter	Effect	Predictor	Outcome	Estimate (s.e.)	p
FA	Direct Effect	Age	Amyloid-β	0.202 (0.061)	0.001
		APOE	Amyloid-β	0.322 (0.113)	0.004
		Amyloid-β	Tau	0.236 (0.063)	<0.001
		Tau	FA	-0.046 (0.016)	0.004
	Total Effect	Age	Tau	0.048 (0.019)	0.013
		Age	FA	-0.002 (0.001)	0.060
		APOE	Tau	0.076 (0.034)	0.023
		APOE	FA	-0.004 (0.002)	0.075
		Amyloid-β	FA	-0.011 (0.005)	0.023
MD	Direct Effect	Age	Amyloid-β	0.202 (0.061)	0.001
		APOE	Amyloid-β	0.322 (0.113)	0.004
		Amyloid-β	Tau	0.236 (0.063)	<0.001
		Age	MD	0.012 (0.004)	0.007
		Tau	MD	0.047 (0.019)	0.015
	Total Effect	Age	Tau	0.048 (0.019)	0.013
		Age	MD	0.014 (0.004)	0.001
		APOE	Tau	0.076 (0.034)	0.023
		APOE	MD	0.004 (0.002)	0.096
		Amyloid-β	MD	0.011 (0.005)	0.040
tNDI	Direct Effect	Age	Amyloid-β	0.202 (0.061)	0.001
		APOE	Amyloid-β	0.322 (0.113)	0.004
		Amyloid-β	Tau	0.236 (0.063)	<0.001
		Tau	NDI	-0.064 (0.020)	0.002
	Total Effect	Age	Tau	0.048 (0.019)	0.013
		Age	NDI	-0.003 (0.002)	0.051
		APOE	Tau	0.076 (0.034)	0.023
		APOE	NDI	-0.005 (0.003)	0.065
		Amyloid-β	NDI	-0.015 (0.006)	0.016

Supplementary Table 2. SEM results of multivariate associations among age, APOE, amyloid-β, tau, and white matter microstructural parameters in DLBs. Abbreviations: SE = Standard errors, APOE = Apolipoprotein E; DLBs = Dementia with Lewy bodies spectrum; DTI = Diffusion tensor imaging; NODDI = Neurite Orientation Dispersion and Density Imaging; PiB = Pittsburgh compound B; SUVr = Standardized uptake value ratios; SEM =

Structural equation model; FA = Fractional anisotropy, MD = Mean Diffusivity; tNDI = tissue-weighted Neurite Density Index.
