# Supplementary material for: Influences of amyloid-β and tau on white matter neurite alterations in dementia with Lewy bodies
Source: NPJ Parkinsons Dis. 2024 Apr 3;10:76. doi: 10.1038/s41531-024-00684-4 (PMC10991290; doi:10.1038/s41531-024-00684-4)
Supplement: Supplementary file 2 — Reporting Summary [file 41531_2024_684_MOESM2_ESM.pdf]

Corresponding author(s): Kejal Kantarci

Last updated by author(s): Jan 1, 2024

Reporting Summary

Nature Portfolio wishes to improve the reproducibility of the work that we publish. This form provides structure for consistency and transparency in reporting. For further information on Nature Portfolio policies, see our Editorial Policies and the Editorial Policy Checklist.

Statistics

For all statistical analyses, confirm that the following items are present in the figure legend, table legend, main text, or Methods section.

n/a	Confirmed	
☐	☒	The exact sample size (*n*) for each experimental group/condition, given as a discrete number and unit of measurement
☐	☒	A statement on whether measurements were taken from distinct samples or whether the same sample was measured repeatedly
☐	☒	The statistical test(s) used AND whether they are one- or two-sided<br>*Only common tests should be described solely by name; describe more complex techniques in the Methods section.*
☐	☒	A description of all covariates tested
☐	☒	A description of any assumptions or corrections, such as tests of normality and adjustment for multiple comparisons
☐	☒	A full description of the statistical parameters including central tendency (e.g. means) or other basic estimates (e.g. regression coefficient) AND variation (e.g. standard deviation) or associated estimates of uncertainty (e.g. confidence intervals)
☐	☒	For null hypothesis testing, the test statistic (e.g. *F*, *t*, *r*) with confidence intervals, effect sizes, degrees of freedom and *P* value noted<br>*Give P values as exact values whenever suitable.*
☒	☐	For Bayesian analysis, information on the choice of priors and Markov chain Monte Carlo settings
☒	☐	For hierarchical and complex designs, identification of the appropriate level for tests and full reporting of outcomes
☐	☒	Estimates of effect sizes (e.g. Cohen's *d*, Pearson's *r*), indicating how they were calculated

Our web collection on statistics for biologists contains articles on many of the points above.

Software and code

Policy information about availability of computer code

Data collection	SAS, version 9.4 (SAS Institute)<br>R statistical software version 4.2.2 (RFoundation)
Data analysis	SAS, version 9.4 (SAS Institute)<br>R statistical software version 4.2.2 (RFoundation), Mplus version 8.10, FSL, and AMICO

For manuscripts utilizing custom algorithms or software that are central to the research but not yet described in published literature, software must be made available to editors and reviewers. We strongly encourage code deposition in a community repository (e.g. GitHub). See the Nature Portfolio guidelines for submitting code & software for further information.

Data

Policy information about availability of data

All manuscripts must include a data availability statement. This statement should provide the following information, where applicable:

- Accession codes, unique identifiers, or web links for publicly available datasets
- A description of any restrictions on data availability
- For clinical datasets or third party data, please ensure that the statement adheres to our policy

Data used in this study are available upon reasonable request.

Research involving human participants, their data, or biological material

Policy information about studies with human participants or human data. See also policy information about sex, gender (identity/presentation), and sexual orientation and race, ethnicity and racism.

Reporting on sex and gender	There were no statistically significant differences between the groups in terms of sex distribution, education years, or APOE status.
Reporting on race, ethnicity, or other socially relevant groupings	We did not consider race or ethnicity as this is beyond the scope of the investigation.
Population characteristics	Participant characteristics are summarized in Table 1. By design, the DLBs and CU groups were comparable in terms of age, with an average age of 68.8 ± 9.2 years (p=0.27). There were no statistically significant differences between the groups in terms of sex distribution, education years, or APOE status. As expected, the DLBs had statistically significantly lower MMSE scores (23.5 ± 5.3) than the CU group (29.2 ± 0.8; p<0.001).
Recruitment	We included consecutive patients with clinically probable DLB 29 who were at mild to moderate clinical stages (n = 32) and those with prodromal DLB (n = 13) 30 to comprise a group of people on the DLB spectrum (n = 45) who were enrolled at the Mayo Clinic Alzheimer's Disease Research Center between February 2018 and October 2021. Clinical diagnosis was established by a consensus committee including behavioral neurologists, neuropsychologists, and study coordinators. An automated greedy match algorithm was used to match 1:1 the CU controls (n = 45) on age and gender from the Mayo Clinic Study of Aging, which is an epidemiologic study of aging in Olmsted County, MN. All participants in the study met specific inclusion criteria, including having multi-shell diffusion data, amyloid-β, and tau PET scans. Exclusion criteria for the DLBs were a history of traumatic brain injury, hydrocephalus or intracranial mass, a history of chemotherapy, head radiation therapy, or substance abuse, and having neurologic or psychiatric disorders other than DLB. The Mayo Clinic Institutional Review Board approved the study. Informed consent for participation was obtained from all patients or a surrogate according to the Declaration of Helsinki.
Ethics oversight	The Mayo Clinic Institutional Review Board approved the study. Informed consent for participation was obtained from all patients or a surrogate according to the Declaration of Helsinki.

Note that full information on the approval of the study protocol must also be provided in the manuscript.

Field-specific reporting

Please select the one below that is the best fit for your research. If you are not sure, read the appropriate sections before making your selection.

☒ Life sciences ☐ Behavioural & social sciences ☐ Ecological, evolutionary & environmental sciences

For a reference copy of the document with all sections, see nature.com/documents/nr-reporting-summary-flat.pdf

Life sciences study design

All studies must disclose on these points even when the disclosure is negative.

Sample size	N = 45 per group. Sample size included all participants with good quality PET imaging and MRI datasets.
Data exclusions	NA
Replication	One of the reviewers enquired about partial-volume corrected PET datasets. These analyses were repeated with partial volume corrected data and findings did not change (provided in the Supplementary Materials).
Randomization	An automated greedy match algorithm was used to match 1:1 the CU controls (n = 45) on age and gender from the Mayo Clinic Study of Aging, which is an epidemiologic study of aging in Olmsted County, MN
Blinding	NA

Reporting for specific materials, systems and methods

We require information from authors about some types of materials, experimental systems and methods used in many studies. Here, indicate whether each material, system or method listed is relevant to your study. If you are not sure if a list item applies to your research, read the appropriate section before selecting a response.

Materials & experimental systems

n/a	Involved in the study
☒	☐ Antibodies
☒	☐ Eukaryotic cell lines
☒	☐ Palaeontology and archaeology
☒	☐ Animals and other organisms
☒	☐ Clinical data
☒	☐ Dual use research of concern
☒	☐ Plants

Methods

n/a	Involved in the study
☒	☐ ChIP-seq
☒	☐ Flow cytometry
☐	☒ MRI-based neuroimaging

Plants

Seed stocks: NA

Novel plant genotypes: NA

Authentication: NA

Magnetic resonance imaging

Experimental design

Design type: NA

Design specifications: NA

Behavioral performance measures: NA

Acquisition

Imaging type(s): Diffusion MRI

Field strength: 3T

Sequence & imaging parameters: All participants underwent a brain MRI protocol on one of three identical Siemens Prisma 3T scanners running VE11 software (uniform versions) equipped with 64-channel receiver head coils. The acquisition protocols were consistent across all three scanners: a magnetization-prepared rapid gradient echo (MPRAGE) sequence with a resolution of 0.8 mm isotropic and a diffusion scan using VE11 Simultaneous Multi-Slice acceleration with adaptive coil combination. The field of view was 232 mm in X and Y and 162 mm in the Z direction with 2.0 mm isotropic voxels for the diffusion scan. The data consisted of 127 volumes, including 13 non-diffusion-weighted images and 114 diffusion-encoding gradient directions. The diffusion-weighted images were evenly spread over the entire spherical shells using an electrostatic repulsion model and were interspersed in time to minimize gradient heating 35. The echo time and repetition time were 71 ms and 3400 ms, respectively. The diffusion scan included b-values of 0, 500, 1000, and 2000 s/mm2.

Area of acquisition: Whole Brain

Diffusion MRI ☒ Used ☐ Not used

Parameters: All participants underwent a brain MRI protocol on one of three identical Siemens Prisma 3T scanners running VE11 software (uniform versions) equipped with 64-channel receiver head coils. The acquisition protocols were consistent across all three scanners: a magnetization-prepared rapid gradient echo (MPRAGE) sequence with a resolution of 0.8 mm isotropic and a diffusion scan using VE11 Simultaneous Multi-Slice acceleration with adaptive coil combination. The field of view was 232 mm in X and Y and 162 mm in the Z direction with 2.0 mm isotropic voxels for the diffusion scan. The data consisted of 127 volumes, including 13 non-diffusion-weighted images and 114 diffusion-encoding gradient directions. The diffusion-weighted images were evenly spread over the entire spherical shells using an electrostatic repulsion model and were interspersed in time to minimize gradient heating 35. The echo time and repetition time were 71 ms and 3400 ms, respectively. The diffusion scan included b-values of 0, 500, 1000, and 2000 s/mm2.

Preprocessing

Preprocessing software	FSL
Normalization	Analyses were performed in native DWI space.
Normalization template	Analyses were performed in native DWI space.
Noise and artifact removal	Head motion and eddy current distortion were corrected using FSL's eddy_cuda, followed by correction of Gibbs ringing 41 and Rician bia.
Volume censoring	NA

Statistical modeling & inference

Model type and settings	Mass univariate
Effect(s) tested	1) Group differences in DTI and NODDI parameteters<br>2) Associations of tau and amyloid with DTI and NODDI parameters

Specify type of analysis: ☐ Whole brain ☒ ROI-based ☐ Both

Anatomical location(s): All ROIs in the the JHU 'Eve' WM atlas, followed by correction for multiple comparisons using FDR.

Statistic type for inference<br>(See Eklund et al. 2016)	NA
Correction	FDR

Models & analysis

n/a	Involved in the study
☒	☐ Functional and/or effective connectivity
☒	☐ Graph analysis
☒	☐ Multivariate modeling or predictive analysis
